# Annotations

**Published:** 1888-04-07

**Authors:** 


### ANNOTATIONS.

**The Presidency of the College of Physicians.**

In the appointment of Sir Andrew Clark, Bart., to the Presidency of the Royal College of Physicians the Fellows have given their suffrages worthily and wisely. The chief merit of any working practitioner of whatever rank is that he honestly and strenuously strives to alleviate suffering, to restore health, and to improve and increase in every direction the general resources of his art. It cannot but be universally admitted that on all these lines Sir Andrew Clark has displayed a conscientious and untiring industry, and a cultured ability of the highest order. Whatever scientific and literary attainments he may possess, and these are neither few nor inconspicuous, he has brought them all to bear on the one duty of understanding, and, whenever possible, helping, his patients. This is surely the most important and the most honourable of all the labours of the physician. The physiologist and the pathologist have each his place, and their work is of a value which cannot be exaggerated. To them it is to a large extent due that medicine is now an honest art, based on a demonstrable science. They are worthy of all the honour which careful and accurate work always commands. In their special domains Sir Andrew Clark has often proved himself a diligent and successful toiler. But it is in the undoubtedly higher fields of actual practice among the sick that he has won his fairest laurels. In the performance of what is often regarded as commonplace daily duty he has displayed an earnestness, a thoroughness, and an uniform capacity which is seldom equalled, and has, perhaps, never been surpassed. The reward which has now been bestowed upon him has come in the full tide of his powers. He is still, and may long hope to be, at his best. His elevation is a distinct encouragement to all other physicians who, whilst searching and exploring every region for the increase and correction of their knowledge of material facts, at the same time devote themselves with a like thoroughness to the acquirement of that practical skill and resource in treatment which the world most needs, and which it must always be the chief distinction of the physician to possess. We sincerely congratulate the College of Physicians on its choice, and Sir Andrew Clark on the distinguished elevation to which, by the suffrages of his peers, he has now been raised.

**Infectious Cases: Temporary or Permanent Hospitals?**

Dr. Benjamin Ward Richardson sounds a decided note in favour of temporary as against permanent hospitals for infectious cases. It is not necessary to say that the opinions of so eminent a sanitarian and original thinker as Dr. Richardson are entitled to serious consideration. Writing on the topic which is now agitating medical and sanitary minds at Nottingham he says: "In the matter of an hospital for infectious cases nothing can be more obsolete than to think of building in such a town as Nottingham one that shall be assumed to last a hundred years. . . For Nottingham at the present time, at a moderate cost, a hospital for infectious cases, capable of meeting every want, can be erected, and may be enlarged or reduced as required, that may be disinfected every day, that may be removed on the shortest notice, if removal be necessary, that may admit of easy improvement from time to time, and that may afford the very best accommodation to the sick within it, without a possibility, if it be managed with judgment, of doing any injury to those who are not in it. As to any difficulties in the construction of such a hospital, they are entirely imaginary, and, indeed, have been already practically met." He describes two temporary hospitals erected some time ago in the stoneyards of the Marylebone parish. The practical working of those revealed certain defects which in future hospitals might easily be remedied. "A gas furnace always alight at the roof of the hospitals, through the flames of which the air could at all times be extracted, and its organic products consumed, was one important desideratum. A method that might very easily have been carried out, of sweeping the walls and ceiling with a brush of flame, was another want. A proper impermeable earthenware surface for the floor, also a very simple improvement, was a third requirement. The one practical fact was that the hospitals served their purpose well, and would have done so for many years had they been required." The difficulty of proper heating Dr. Richardson regards as entirely visionary. "With two walls of iron, or a wall of iron on the inside and a wall of wood on the outside, and a good air space between the walls, into which warm air can be admitted, the most perfect and equable warming may be secured. . . . Nottingham is an important place to set an example. What she does will be followed. . . . If she now goes to the absurd expense of putting up a hospital for infectious disease, destined, like a Norman fortress, to last for any number of years, she will simply perpetuate a series of great evils." The leaders of sanitary opinion at Nottingham and elsewhere will be well advised to give careful heed to these weighty and measured utterances.

**Mr. Walter Besant's Craze about Women.**

Certain African tribes make drudges of their women, sending them to sow and weed and reap the fields, whilst they stay at home, or lie under the shade of the trees drinking Pombé, and otherwise enjoying themselves. That is not a very chivalrous way of doing; but it at least recognises that woman is of some value, inasmuch as she is of some use. More civilised people, like ourselves, are accustomed to regard woman as the companion, the equal, and the helper of man; the wife being looked upon as the one friend on whom reliance can be placed at all times, the one yokefellow who, whatever betide, will always be in her place, always loyal, always helpful. Mr. Walter Besant, quite unconsciously we hope, is all for reducing women to an altogether lower level He thinks they should never be compelled to work for their own livings like the other sex, but should always have husbands to work for them; and when husbands cannot be found, fathers, or brothers, or other near relatives should be responsible for the assured supply of their daily needs. Is Mr. Besant really serious in this unnatural and impossible suggestion? We admit, as fully as Mr. Besant, or any other deeply sympathetic spirit, the strain and the worry and the failure which happen to thousands of women in this country annually; but the very same strain and worry and failure happen to men. Does Mr. Besant propose that men too should be provided for, all except those of unusual genius and practical capacity? The simple truth is that in this country the population is too large; the workers are more than the work, and so wages of all kinds are reduced, and thousands of both sexes suffer. But surely the way to do is not to diminish the number of workers by converting some of them into idlers, and so increasing the strain upon the others—husbands, fathers, brothers, and so on. The only result of that would be that the same amount of wages would be earned, but earned by fewer workers, and at greater expenditure of nervous and vital energy. Besides, as surely as any person, man or woman, becomes an idler, so surely does he or she deteriorate in character, and become more frivolous, more exacting, and less capable of understanding the difficulties and anxieties of those who bear the stern burdens of modern life. The gospel which proclaims that men should seek for women an existence of careless ease and ignorance of the true conditions of life's battle may sound very amiable but is very silly and pernicious. The best women will scorn it, and be ashamed that it should be promulgated in their interests. The real gospel to teach women is that the more they try to understand the labours and difficulties of life, the more they enter into and share them with their fathers, brothers, and husbands, the more they try to be fighting companions and working partners, the better it will be for their body and mind, the better for their families, the better for the world at present, and the better for succeeding generations. Life at best is a hard battle for many, but it is easiest and most victorious when all true spirits take their share in the conflict.

